# The Role of Attachment in Poly-Drug Use Disorder: An Overview of the Literature, Recent Findings and Clinical Implications

**DOI:** 10.3389/fpsyt.2019.00579

**Published:** 2019-08-27

**Authors:** Michaela Hiebler-Ragger, Human-Friedrich Unterrainer

**Affiliations:** ^1^Department for Psychiatry and Psychotherapeutic Medicine, Medical University of Graz, Austria; ^2^Center for Integrative Addiction Research (CIAR), Gruener Kreis Society, Vienna, Austria; ^3^Department of Religious Studies, University of Vienna, Vienna, Austria

**Keywords:** substance use disorder, attachment, emotion regulation, treatment, polydrug use disorder

## Abstract

**Background:** Substance use disorders (SUDs) represent a worldwide epidemic with extensive costs to the individual and to society. Occasionally described as an attachment disorder, they have been linked to various impairments in self-regulation and social functioning. However, while there have been significant advances in the development and validation of treatment strategies for SUD in recent years, the components of these treatment approaches have yet to be fully explored. The characteristics of polydrug use disorder (PUD) especially need to be addressed in more detail, as this diagnosis is highly common in individuals seeking treatment, while simultaneously being associated with poor treatment success.

**Aim and Scope:** This review aims at further exploring the relevance of attachment in PUD and its treatment. To this end, this review provides a concise summary of relevant theories on the development and treatment of SUD in general, including related parameters of attachment, emotion regulation, and neuroscience. Furthermore, several studies focused specifically on PUD are described in more detail. These studies explored the connections between attachment, personality structure, primary and higher emotions (including spirituality), as well as structural and functional neural parameters in inpatients with PUD as well as in healthy controls. Most notably, the described studies highlight that insecure attachment and impairments in personality structure are present in inpatients with PUD. In addition, these characteristics are paralleled by extensive impairments in white matter integrity, especially in tracts connected to facets of emotion regulation.

**Conclusions:** Based on our findings, we emphasize conceptualization of PUD as an Attachment Disorder, on a behavioral as well as on a neural level. Furthermore, we point out the importance of an integrated bio-psycho-social approach in this research area. Consequently, future studies might more closely focus on the influence of attachment-based interventions on emotion regulation abilities as well as a potentially related neuroplasticity. Neuroplastic changes, which are still rather unexplored, might represent important parameters for the assessment of treatment outcomes especially in long-term SUD treatment.

## Introduction

As it has been suggested that individuals with polydrug use disorder (PUD) differ from individuals with other substance use disorders (SUDs) (1) and that they consequently may need different treatment settings (2), we dedicated five studies to the exploration of attachment and related parameters in inpatients with PUD. In this review, we summarize current theoretical models and empirical results related to the conceptualization of addiction as an attachment disorder before discussing our results on PUD and their implications for future research and clinical practice.

SUDs represent a worldwide public health problem [e.g., Ref. (3)]. As the social, occupational, mental, and physical problems connected to these disorders often persist even after abstinence is achieved, the direct and indirect costs of SUD to the individual and to society are extensive (4). Polydrug use is especially common among drug users worldwide (1). Furthermore, most individuals in treatment for SUD report a PUD (2). As previous studies have suggested numerous differences between PUD and Mono-SUD regarding personality (e.g., impulsivity) as well as etiological (e.g., emotional neglect in childhood) factors (5), it has been suggested that individuals with PUD might even need different treatment settings (2). However, while countless studies have focused on understanding the multifactorial and complex nature of different SUD in order to optimize prevention and treatment, “many challenges remain to understand and treat drug addiction” (p.1) (6).

Bowlby (7) already noted that insecure attachment patterns can help to explain “the many forms of emotional distress and personality disturbances, including anxiety, anger, depression, and emotional detachment, to which unwilling separations and loss give rise” (p. 201). Accordingly, insecure attachment patterns have been extensively discussed as contributing to different facets of personality pathology (8, 9) as well as a large number of other psychiatric diseases, including affective disorders in addition to SUD (10).

**Figure 1:**
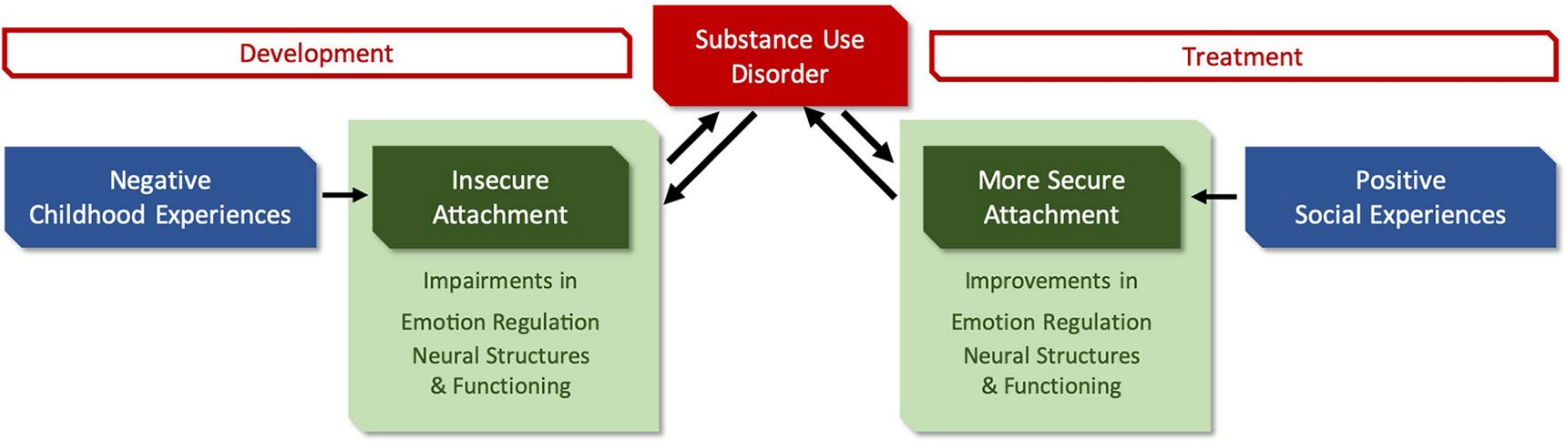
Brief overview of the influence of attachment on SUD. The figure details the role of attachment patterns that form through social experiences, as well as related parameters on the development and treatment of substance use disorders (SUDs).

Importantly, attachment theory offers the great advantage of not only informing our understanding of the development of psychopathology but also of the development of mental health and well-being. **Figure 1** gives a short overview regarding the mechanisms underlying the development and treatment of SUD that will be described in this review.

Consequently, this review aims to describe how insecure attachment (developed in response to negative childhood experiences) leads to diverse vulnerabilities (e.g., impairments in emotion regulation and neural parameters) that may contribute to the development of SUD and that are in turn influenced by the dynamics of SUD. On the other hand, positive social experiences during treatment may promote the development of more secure attachment (including for example improvement in emotion regulation) that supports an increased independence from psychoactive substances (see **Figure 1**).

In this paper, we will therefore first provide a concise summary of the background relevant for our research on PUD (i.e., attachment theory and its relationship with personality structure, conceptualization of SUD and its relation to attachment in general, neural parameters underlying attachment and emotion regulation, treatment of SUD with a focus on the therapeutic community). Consequently, we will discuss five studies focused on inpatients with PUD undergoing treatment in a therapeutic community setting. Lastly, we will discuss the results of these studies in relation to current research and theoretical models with a special focus on attachment-related parameters (e.g., emotion regulation) and treatment approaches.

### Attachment Theory

As “adaptations to early experiences set the stage for negotiating later experiences” (11), the development of adult psychopathology has to be considered in light of the interactions between earlier experiences, the resulting adaptation, and current contextual parameters (12). Furthermore, most theories of development include the fundamental concept that social relationships both influence and are influenced by the development of psychopathology: Therein, secure attachment is generally thought to act as a protective factor, while insecure attachment is thought to increase the vulnerability for psychopathology [for an overview, see Ref. (13)]. Importantly, however, the possible influence of attachment always has to be considered in the context of other risk factors (14–16) as pathology is unlikely to be caused by a single risk factor.

According to attachment theory, attachment is not a mere secondary drive but has to be seen as a fundamental primary motivation with its own dynamics (17). As it—ideally—establishes a “secure base” from which the individual can explore the world as well as a “safe haven” to retreat to in times of distress (18), the attachment system has an important impact on everyday person–environment interactions (19).

#### Dynamics and Styles of Attachment

Attachment theory (17, 18, 20) differentiates between a secure attachment style that is established through a sensitive, supportive, and caregiving environment and insecure attachment styles that are the result of an inconsistent, insensitive, or dismissive attachment figure. Mikulincer and Shaver (21) differentiate between two basic attachment dimensions: Anxious attachment and avoidant attachment. Accordingly, secure attachment (low anxious and avoidant attachment) allows the individual to deal with stressful experiences by relying upon mental representations of previously received support or by actively seeking support in the present (22). Individuals with high levels of anxious attachment, characterized by the use of hyperactivating strategies, actively demand support even though they may feel unworthy of love (21). For individuals with high levels of avoidant attachment, characterized by the use of deactivating strategies, they pride themselves on their self-reliance, which in turn can lead to a denial of personal imperfections and weaknesses (23).

Lastly, fearful attachment is defined by high levels of anxious attachment and avoidant attachment (21, 24). While “normal” samples mostly contain individuals with secure attachment (about 70%) (25), individuals with extremely high scores on both anxious attachment and avoidant attachment (i.e., fearful attachment) are most likely to be found in abused or clinical samples [for an overview, see Ref. (26)]. Fearful attachment can furthermore be described as disorganized, since individuals with this attachment style seem unable to develop an organized strategy, whether to rely on hyperactivating or deactivating behaviors to get their attachment needs met [e.g., Ref (22)]. Therefore, rather than enacting and habituating reliable clinging/whining/attention-seeking (hyperactivating) or withdrawing/dissociative (deactivation) behaviors, they simply erupt into some kind of behavior in an effort to relieve stress and may even combine hyper- and deactivation strategies into odd and ineffective responses.

#### Attachment and Personality Structure

Bowlby, describing the long-lasting effects of attachment across the lifespan (7), has already named early attachment experiences as an important factor influencing personality structure. Essentially, while the internal working models defined by attachment theory have a strong focus on the content and behavioral consequences of mental representations, the concept of personality structure extends this model by adding the complexity of their structural organization and integration. Therefore, individuals with similar attachment patterns might vary regarding the level of integration and differentiation of their internal working models (27, 28). In general, however, more insecure attachment patterns seem to be associated with lower levels of structural integration (i.e., more impairments in personality structure) (29).

A good structural integration is defined by a relatively autonomous self that shows stability as well as flexibility when adequately processing impulses, emotions, and conflicts (30). A moderate structural integration is defined by a tendency towards overcontrolling as well as an increased occurrence of self-destructive impulses. A low structural integration is defined by impaired regulatory functions, which leads to repetitive flooding with intense negative affect as well as (self-) destructive impulses (30). Lastly, a disintegrated structure is defined by the central fear that the sense of self vanishes due to a symbiotic merging of the self and objects (30). Consequently, patients with a low level of structural integration seem to be more likely to experience psychotic symptoms (31), to have a longer duration of mental illness (32), and to be recommended psychiatric instead of psychotherapeutic treatment (33). Conversely, both patients and therapists rate a higher level of structural integration as advantageous for the success of treatment and a change in symptoms (32, 34).

#### Attachment and Emotion Regulation

Various forms of SUD, including PUD, have been linked to impairments in the cognitive control of emotions [for an overview, see Ref. (35)].

Importantly, deficiencies in emotion processing and regulation are a known “liability spectrum that underlies many different mental disorders” (p. 154) (36). Developing the capacity for healthy interpersonal affect regulation requires the development of a secure attachment style, as individuals with secure attachment are willing and able to acknowledge and communicate their emotions (37). Therefore, the primary function of adult attachment relationships may be seen in the social regulation of emotions (38).

Consequently, the use of psychotropic substances has been connected to anxious attachment (39, 40), avoidant attachment (41, 42) and disorganized attachment (39, 43, 44). This indicates that the deprivation of developmental needs generally can result in vulnerabilities that in turn lead to misguided attempts at self-repair, leaving the individual “constantly searching for something ‘out there’ that can be substituted for what is missing ‘in there’” (p. 7) (45). This coincides with the psychodynamic point of view that substance abuse “represents a failure to negotiate the transition from helplessness to competence in the social world” (p. 2004) (10). Importantly, the conceptualization of SUDs as an “Attachment Disorder” does recognize that SUDs are not a one-dimensional phenomenon: While substance abuse is initially used by the individual to deal with difficulties in interpersonal relationships, it consequently gradually increases the impairments in “an already fragile capacity for attachment” (p. 2) (45).

#### Neural Structures Related to Attachment and Emotion Regulation

Although numerous neurobiological studies in the past few decades focused on attachment in nonhuman animals, such research in humans is relatively limited (46). Consequently, neural circuits underlying attachment are as yet relatively unknown (47). In addition, one has to keep in mind that the attachment behavioral system is highly unlikely to be related to a singly, dedicated attachment circuit, as this higher-order construct makes use of multiple subsystems (e.g., emotion, memory, perception, motivation) (46). In light of these multiple subsystems involved in attachment, it may even be suitable to “think of the entire human brain as an attachment system” (p. 244) (46).

As one of the most important brain structures associated with emotion (48), the amygdala reacts to both unconditioned and conditioned signs of threat (46) and is highly sensitive to facial social signals (49, 50). Together with the hippocampus that is involved in the formation of associations between internal states and environmental stimuli (51), the amygdala consequently enables the identification and consolidation of important interactions with attachment figures as well as emotionally salient situations (46).

Strongly connected to these brain structures, the prefrontal cortex plays an important role in motivation as well as emotion regulation (46, 52, 53). In detail, the prefrontal cortex seems to be connected to attachment through the encoding of “automatic” (conditioned through threat related stimuli) responses to the attachment figures as well as the “effortful” modulation of cognitive operations involving the attachment figures (46).

While secure attachment is generally thought to be associated with less reactivity to distress, insecure attachment seems to be connected to increased neural activation throughout the brain under conditions of distress (e.g., pain or threat) (46). Furthermore, individuals with avoidant attachment seem less able to profit from the presence of others in times of distress but rather tend to perceive them as an additional burden (46). Among these processes, social affect regulation can be seen as a bottom-up mechanism, while affect regulation without support from others can be seen as a top-down mechanism. These top-down mechanisms include effortful cognitive and attentional emotion regulation strategies, such as suppression or cognitive reappraisal, that rely heavily on the prefrontal cortex (46).

### Substance Use Disorders

#### Definition and Diagnostic Criteria

In the literature on SUD, various terms are used to describe the relation between a psychotropic substance and its user. Consequently, a SUD can be described as chronic, relapsing disorders defined by 1) the compulsive seeking and taking of psychotropic substances, 2) a loss of control regarding these behaviors, as well as 3) the emergence of withdrawal symptoms that include negative emotions (e.g., irritability, anxiety) when these behaviors are unfruitful.

While similar criteria for SUD can be found in Diagnostic and Statistical Manual of Mental Disorders IV (DSM-IV) (54) and International Classification of Diseases in 10th revision (ICD-10) (55), conceptual and diagnostic changes have been made in the DSM-V (56): Here, the criteria for substance abuse and substance dependence have been merged into one continuum of SUDs, ranging from mild to moderate to severe, based on the number of criteria met.

#### Development of Substance Use Disorders

Diverse pathways and multiple, interacting processes may lead to SUD, with the individuals abusing or dependent on one or more of these substances consequently representing a highly heterogeneous group: Differences might be present, for example, in social development, comorbidity, neurobiological processes and genetics (57). The importance of applying a developmental perspective—as provided for example by attachment theory—to the study of SUD is underlined by various aspects: Epidemiological data reveal characteristic age-related trajectories for SUD, progressing from the typical onset of substance use and SUD during adolescence to peak rates in young adults and to a decline in later life (58).

Regarding the development of a PUD, developmental progression may not only apply to the stages of use—ranging from occasional use to dependence—but also across substances: For example, individuals often seem to progress from “gateway” substances (e.g., tobacco, alcohol, cannabis) to the use of other psychotropic substances (59–62). This progression might be attributed to several factors, including a common propensity to use psychotropic substances, a sensitization for the use of other substances due to the use of a previous substance, or a connection to a social network that promotes the use of several substances (61, 63, 64).

#### Neural Parameters of SUD

Several studies using magnetic resonance imaging (MRI) techniques have reported altered brain morphology in various SUDs [for an overview, see Ref. (65)]: Regarding gray matter, impairments have particularly been reported in the frontal lobes, the amygdala, and the insula. Regarding white matter, impairments have especially been reported in the genu and the corpus callosum as well as in prefrontal regions. In general, these impairments seem to be relevant for various cognitive dysfunctions relevant in SUD (e.g., increased impulsivity and impaired executive functions) [for an overview, see Ref. (65)]. However, there is still some debate as to how and to what extent SUDs are connected to impairments in white matter integrity (66, 67). For example, impairments in self-regulation and executive functions, connected to dysfunctions or pathologies in the frontal lobes, represent a risk factor not only for SUD but several psychiatric disorders (68). Regarding white matter tracts, a healthy development is necessary for an efficient communication between brain regions, higher order cognitive functioning, as well as several complex behaviors (69). Consequently, substance abuse is likely particularly harmful during adolescence, when white matter is still developing (66, 70–72).

In general, neural impairments connected to SUD seem to be especially prevalent in the above described structures related to attachment and emotion regulation (e.g., in the amygdala or the prefrontal regions).

#### Treatment of SUD

Most specialists for the treatment of SUD (intuitively) recognize the importance of attachment in addiction, independent of whether interpersonal problems are the cause or the consequence of drug use (45). However, before an attachment to treatment (e.g., a therapeutic alliance) can be established, individuals with a SUD must first become detached from the substances they abuse (45). Therefore, the consideration of attachment theory in the treatment of SUD highlights the importance of the therapeutic alliance (73).

While there have been significant advances in the development and validation of psychosocial treatment strategies for SUD in the past few decades, the parameters for the success of these approaches have yet to be fully explored. A meta-analytic review by Dutra and colleagues (74) found moderate effect sizes for psychosocial treatments, but these effect sizes varied considerably dependent on the SUD and the treatment strategy under study; although individuals with Cannabis Use Disorder appeared to profit considerably from psychosocial interventions, individuals with PUD seem to profit the least. Drop-out rates were high (around one third) across all psychosocial interventions, but approximately the same percentage of participants achieved posttreatment and/or clinically significant abstinence (74).

Since the establishment of opioid substitution in the 1960s, this treatment strategy for opioid use—that is highly prevalent in PUD (75)—went hand-in-hand with psychosocial interventions. Accordingly, international clinical guidelines list psychosocial rehabilitation as crucial in this area (76). However, while several randomized controlled trials and systematic reviews conclude that opioid substitution is just as effective or even more effective when provided on its own, some large outcome studies have concluded that treatment providers with a higher frequency and quality of psychosocial interventions are better able to achieve positive outcomes [for an overview, see Ref. (76)].

Contrary to the classic psychodynamic developmental model, attachment-oriented treatment does not equate mental health and maturity with independence (45, 73). In line with Bowlby (20), normal development is seen as a movement from immature dependence towards mature interdependence and mutuality (73). Consequently, group therapy has been an important component of the treatment for SUD ever since the establishment of Alcoholics Anonymous (AA) in the 1930s (73). This can be attributed to the interpersonal conception of group therapy (human beings are always considered as social and as being situated in relation to others) that is more likely to promote attachment than other treatment strategies (73).

Interestingly, spirituality is also considered to be a helpful factor in the treatment of SUD (77). While it is closely connected to the AA program, it has also been incorporated in other treatment strategies (78, 79). This is not surprising, given that the relationship between believers and a higher power (e.g., God or other divine figures) frequently fulfils the criteria of an attachment bond and can consequently be assumed to enable similar psychological advantages (80). As the sense of having a secure attachment bond with a higher power is associated with higher spiritual well-being (81), spirituality can be conceptualized as the “ability to experience and integrate meaning and purpose in existence through a connectedness with self, others or a power greater than oneself” (p. 117) (82). Consequently, more secure attachment seems to be related to lower levels of mood pathology in general and in individuals with SUD (81, 83).

As an extension of group therapies, therapeutic communities were established in the 1960s as long-term (several months) residential programs for individuals with SUD (84). According to their conceptual groundings, the extent of impairments in psychological dysfunction and social deficits is more important than a certain pattern of drug use. Considering “community as method,” the most important psychological treatment goals are to restructure the negative patterns of behavior, thinking and feeling using self-help, mutual self-help, and social learning (84). A long-term stay within this caregiving, abstinence promoting environment should encourage alternative emotional experiences and, consequently, stimulate a kind of subsequent maturation of former inadequate attachment patterns (45).

### Implications of PUD

While several studies report high levels of PUD in patients with SUD as well as a greater SUD severity in patients with PUD (e.g., 2, 85), comparatively few studies consider a wide range of drug types and/or classes, thereby neglecting the issue of polydrug use and PUD (86). Furthermore, the different definitions of polydrug use applied in SUD research often make it difficult to compare studies (87). These tendencies may lead to research results that provide little relevant information for clinicians involved in SUD treatment programs. On the other hand, explicit evaluations of polydrug use could have a high clinical as well as public health relevance (60).

In general, especially adolescents with self-perceived low social standing and lower parental socioeconomic status seem to be at risk to develop this pattern of drug use (88). Therein, polydrug use seems to be more prevalent in young men than young women (89, 90) and comorbid mental disorders seem to be more prevalent in young adults with PUD compared to those with another SUD (91). Importantly, while polydrug use is highly prevalent in individuals with opioid use disorder, individuals with this pattern of drug use also show a high prevalence of comorbid posttraumatic stress disorder (PTSD) (75). Therein, PTSD and PUD may be connected by “an ‘additive’ self-medication model” (p. 39) (92). In addition, the number of polysubstance opioid overdoses seems to be increasing in certain areas (93) and SUD persistence rates seem to be consistently higher in PUD than in other SUD (94). These recent findings further underline the need for addressing polydrug use and related characteristics in SUD research and clinical practices.

While this review focuses mainly on PUD, the reported mechanisms related to attachment and emotion regulation as well as their neural correlates may be largely seen as liabilities relevant to SUD in general. In line with this, recent findings suggest that treatment strategies should target these broader liabilities instead of focusing on specific SUD (95). However, several studies also highlight the need for a closer examination of the characteristics and treatment requirements of individuals with PUD (92, 96, 97).

## Research Focused on Poly Drug Use Disorder

Based on the above described theoretical and empirical background, our research group conceptualized five studies—three of which used (f)MRI—that aimed at further exploring attachment and related parameters in PUD. A concise overview on the methods and results of each study can be found in **Table 1**. Additional information on the presented studies (e.g., statistical analyses, sample characteristics) can be found in the related publications (98–102) or obtained from the authors.

**Table 1:** Methods and results of studies on PUD.

	Sample		Methods		Results
	SUD	Controls	Questionnaires and tests	(f)MRI parameters	
**Attachment and neural parameters**					
Study 1 (100)	PUDa: *n* = 18 PUDm: *n* = 15	*n* = 16	AAS, BSI-18, NEO-FFI, IPO-16, WPT	White Matter: FA, RD	PUD showed - more insecure attachment, - more impairments in personality structure, - more neuroticism and agreeableness. In addition, PUD showed - reductions in FA and - increases in RD in mainly the same white matter tracts.
Study 2 (99)	PUD: *n* = 19	RUC: *n* = 20 NUC: *n* = 20	AAS, MI-RSWB, BANPS WPT	White Matter: FA ROIs: SLF, SCR	PUD showed - more insecure attachment, - more negative primary emotions. In addition, PUD showed - reductions in FA.
Study 3 (101)	PUD: *n* = 18	n = 16	RIT, ERQ, OPD-SQ, BSI-18, WPT	RGT	PUD showed - more insecure attachment, - more impairments in personality structure, - more mood pathology, - poorer emotion regulation skills. No group differences in reappraisal-related neural activation were found.
***Attachment and treatment adherence***					
Study 4 (98)	AUD: *n* = 66 PUD: *n* = 57	n = 114	ASQ, BPI	–	AUD or PUD showed - more aspects of borderline personality structure, - different attachment patterns than CG. No differences could be observed between AUD and PUD inpatients.
Study 5 (102)	AUD: *n* = 66 PUD: *n* = 57	–	ASQ, BPI At treatment entry and after six weeks	–	Inpatients with more “Confidence in Self and Others” were more likely to drop out of treatment.

PUDa, inpatients with a PUD that were abstinent; PUDm, inpatients with a PUD undergoing maintenance therapy; AAS, Adult Attachment Scale; BSI-18, Brief Symptom Inventory; NEO-FFI, Neuroticism Extraversion Openness Five Factor Inventory; IPO-16, 16-Item Inventory of Personality Organization; WPT, Wonderlic Personnel Test; FA, Fractional Anisotropy; RD, Radial Diffusivity; RUC, controls with recreational drug use; NUC, non-drug-using controls; MI-RSWB, Multidimensional Inventory for Religious/Spiritual Well-Being; BANPS, Brief Affective Neuroscience Personality Scale; ROI, Region of Interest; SLF, superior longitudinal fasciculus; SCR, superior corona radiata; RIT, Reappraisal Inventiveness Test; RGT, Reappraisal Generation Task; ERQ, Emotion Regulation Questionnaire; OPD-SQ, OPD Structure Questionnaire; AUD, Alcohol Use Disorder; ASQ, Attachment Style Questionnaire; BPI, Borderline Personality Inventory. Additional information on the presented studies (e.g., statistical analyses, sample characteristics) can be found in the related publications (98–102) or obtained from the authors.

### Attachment and Neural Parameters

Regarding attachment and neural parameters in PUD, two studies focused on the relevance of potential impairments in white matter integrity, while one study explored neural activation patterns during a novel emotion regulation task. The aim was to gain new insights into the bio-psycho-social interactions underlying PUD.

#### Study 1

As previous studies indicated that a drug substitute (e.g., Methadone) could artificially alter the attachment status so that insecure individuals would appear secure (10, 103), our first study (100) explored whether inpatients with PUD, who were either abstinent or in maintenance treatment, differed regarding white matter structure (assessed by means of diffusion tensor imaging) as well as cognitive ability, attachment style, and personality/mood pathology.

##### Methods and Results

In the first study (100), the sample of 49 men included inpatients with a PUD who were either abstinent (PUDa; *n* = 18) or undergoing maintenance therapy (PUDm; *n* = 15) as well as a control group of healthy students (CG; *n* = 16). In addition to the *Adult Attachment Scale* (AAS) [Ref. (104, 105), and the *Brief Symptom Inventory* (BSI-18) (106), participants completed the *Neuroticism Extraversion Openness Five Factor Inventory* (NEO-FFI) (107) assessing Extraversion, Agreeableness, Conscientiousness, Neuroticism, Openness to Experience, and the *16-Item Inventory of Personality Organization* (IPO-16) (108) assessing Identity Diffusion, Primitive Defense, and Reality Testing as potential impairments in personality structure. Lastly, the *Wonderlic Personnel Test* (WPT) was used as a rough screening instrument for intelligence (109). White matter integrity was assessed through diffusion tensor imaging (DTI) that is based on the directionality and rate of diffusion of water within tissue. Consequently, a higher fractional anisotropy indicates for example that diffusion is restricted by the myelin sheaths of axons (110).

Regarding personality characteristics, PUD showed a higher amount of insecure attachment, a higher total amount of impairments in personality structure, indicating a higher risk for personality disorders (108), as well as higher amounts of neuroticism and agreeableness. Regarding white matter integrity, group differences in FA and radial diffusivity (RD) were generally more pronounced between CG and PUDa than between CG and PUDm, with both clinical groups showing widespread reductions in FA and increases in RD mainly in the same white matter tracts (mostly the superior corona radiata and the superior longitudinal fasciculus of the right hemisphere) (100). In general, lower FA and higher RD indicate a higher probability for white matter impairments (111). Interestingly, more insecure attachment and more impairments in personality structure were related to lower FA (*r* = -.36 to -.41) and higher RD (*r* = .31; all *p* < .05) over all participants.

##### Discussion of Results

The results of the first study (100) indicate that impairments in white matter structure are present in inpatients with PUD and that these impairments are paralleled by a higher amount of mood and personality pathology. In line with the conceptualization of SUD as “Attachment Disorders” (112) and in accordance with previous work (42), substituted inpatients with PUD seem to show the highest amount of anxious attachment.

Contrary to our assumptions, no significant differences in white matter integrity between abstinent and substituted inpatients with PUD were found. However, differences in white matter parameters were more pronounced between abstinent inpatients with PUD and healthy controls than between substituted inpatients with PUD and healthy controls (100). This may indicate that white matter integrity deteriorates more under abstinence, as the brain struggles to regain homeostasis (100). As impairments in the superior corona radiata and the superior longitudinal fasciculus have also been observed in adolescent substance abusers, they may be partly premorbid or a very early occurrence in SUD (66). Furthermore, impairments in the superior corona radiata and the superior longitudinal fasciculus appear to be linked with impaired decision-making (113), while impairments in the superior corona radiata can also be linked to higher risk taking in adolescents (69).

#### Study 2

In the second study (99), we focused on the superior corona radiata and the superior longitudinal fasciculus, as deficiencies in these tracts have been linked to SUD in several studies (66, 100, 114). In addition, we hypothesized that higher amounts of existential fear and despair would be connected to more insecure attachment and decreased spiritual well-being in inpatients with PUD (115). Furthermore, following the concept of a severity continuum in SUD (56), we differentiated between non-drug-using controls, recreational drug-using controls, and inpatients with PUD.

##### Methods and Results

In the second study (99), the sample of 59 men included inpatients diagnosed with PUD (PUD; *n* = 19) as well as controls with recreational drug use (RUC; *n* = 20) and non-drug-using controls (NUC; *n* = 20). All participants completed the *Adult Attachment Scale* (AAS) [Ref. (104, 105), the *Multidimensional Inventory for Religious/Spiritual Well-Being* (MI-RSWB) (116) and the *Wonderlic Personnel Test* (WPT) (109) as well as the *Brief Affective Neuroscience Personality Scale* (BANPS) (117) assessing the primary emotions SEEKING, SADNESS, FEAR, ANGER, CARE, and PLAY (118).

Regarding behavioral parameters, PUD showed higher levels of attachment related Anxiety than NUC and RUC as well as higher levels of negative primary emotions than NUC. No differences were found regarding the other variables. To explore possible connections between the behavioral parameters and white matter integrity, a regions-of-interest (ROIs) analysis including the superior longitudinal fasciculus and the superior corona radiata of both hemispheres focused on fractional anisotropy (FA), the most widely used DTI parameter (111). Here, PUD showed a lower FA compared to NUC and RUC in the right and left superior longitudinal fasciculus as well as a lower FA compared to NUC in the right and left superior corona radiata. Furthermore, FA in the right superior corona radiata was related to more secure attachment (*r* = .58) and less FEAR (*r* = -.46; both *p* < .05) (99).

##### Discussion of Results

While the second study (99) also supports the presence of white matter impairments and insecure attachment in inpatients with PUD, some additional insights could be gathered, as increased levels of certain primary emotions also seem to be connected to diminished white matter integrity. As in previous research (119, 120), inpatients with PUD in this study demonstrated a higher amount of ANGER, FEAR, and SADNESS compared to non-using controls (99). However, no differences were found regarding SEEKING, which previously has been theorized to be pathologically abridged in SUD (121, 122). This may be attributed to the fact that the inpatients with PUD in this study were enrolled in a therapeutic community (123, 124) during data acquisition. This treatment approach is theorized to act like a substitution drug, thereby balancing the abridged SEEKING dimension that would otherwise heighten drug craving and the possibility of relapse (121, 125). In addition, the high level of SADNESS in inpatients with PUD may underline the close connection between SUD and depression (10). The tentative connections between attachment, primary emotions, religious/spiritual well-being, and white matter integrity in inpatients with PUD that were found in this study (99) are in line with the notion of including religious/spiritual aspects in addiction treatment. As stated before, this may allow for more secure attachment experiences and could consequently increase the ability for emotion regulation (45, 115, 124).

#### Study 3

In the third study (101), we aimed to generate new information regarding impaired emotion regulation abilities in SUD by the exploratory use of an fMRI paradigm focusing on cognitive reappraisal. This strategy refers to a deliberate re-interpretation in order to modulate emotional impact (126).

##### Methods and Results

The third study (101) tested 34 right-handed men, divided into two groups, one clinical inpatient group (PUD; *n* = 18) diagnosed with PUD and one group of healthy controls (HC; *n* = 16) who reported very little or no experience with illegal substances. Cognitive reappraisal capacity was assessed outside the scanner with the Reappraisal Inventiveness Test (RIT) (127) as well as with the similar Reappraisal Generation Task (RGT) during fMRI: In each test, subjects are instructed to empathize with anger-eliciting situations and to consequently generate different reappraisals in order to downregulate anger. In addition, participants completed the *Emotion Regulation Questionnaire* (ERQ) (128), German version by Abler and Kessler (129), the *OPD Structure Questionnaire* (OPD-SQ) (130) assessing impairments in personality structure with four dimensions (131) that each comprises a self-related and an object-related subdomain: 1) Perception; 2) Regulation; 3) Communication; 4) Bonding, as well as the *Adult Attachment Scale* (AAS) [Ref. (104, 105), the *Brief Symptom Inventory* (BSI-18) (106), and the *Wonderlic Personnel Test* (WPT) (109).

Group comparisons revealed several differences with generally large (*eta* *^2^* > .14) effect sizes (132) between PUD and HC: PUD reported more impairments in personality structure, mood pathology, and insecure attachment. Concerning emotion regulation, PUD reported a less frequent use of reappraisal but a more frequent use of suppression. Regarding cognitive reappraisal, PUD showed lower fluency and flexibility of ideas as well as more induced anger than HC. In line with this, their reappraisals during fMRI were rated as less effective than those of HC. Regarding the reappraisal-related neural activation, remarkably similar patterns were observed for both PUD and HC: They included a rather left-lateralized network of inferior, superior, and middle frontal gyri, supplemental motor areas, as well as pre- and postcentral gyri.

A consequent conjunction analysis on voxels significantly activated in PUD and HC showed in more detail that both groups activated the left inferior and superior frontal gyri, the right cerebellum, as well as the right middle temporal cortex. No group differences in neural activation were found.

##### Discussion of Results

The results of the third study (101) not only underlined our previous findings of insecure attachment and impaired personality structure in SUD but also highlighted the prevalence of impaired emotion regulation abilities in PUD. Although we did not find the expected differences in neural activation patterns during cognitive reappraisal between inpatients with PUD and healthy controls, the pattern of neural activation assessed for both groups highlights the crucial role of the frontal cortex and therefore of executive functions in this emotion regulation strategy (127, 133). Considered together with the poorer behavioral results in cognitive reappraisal in inpatients with PUD, the discrepancy between neural and behavioral results may point towards a third parameter connecting these two levels (101): As our previous two studies (99, 100) found extensive white matter impairments in inpatients with PUD, efforts in cognitive reappraisal could generate the required activation in gray matter structures in inpatients with PUD, but white matter impairments may prevent an adequate interaction between these gray matter structures, which could result in a lower capacity for cognitive reappraisal. In addition, as the contrary strategies underlying different types of insecure attachment—hyperactivating strategies in anxious attachment and deactivating strategies in avoidant attachment (134)—appear to be connected to different or contrary patterns of neural activation during emotion regulation (135), a mixture of these patterns could mask possible differences to healthy controls (101). Importantly, the various possible mechanisms of cognitive reappraisal in SUD need to be explored in more detail in future studies, as reappraisal may be directed at the meaning or the self-relevance of a potentially emotion-eliciting situation in order to increase or decrease negative or positive emotions (136). Furthermore, there is some indication that cognitive reappraisal is only adaptive when dealing with uncontrollable stress (where the only option is self-regulation) but not controllable stress (where the situation can be influenced) (137).

### Attachment and Treatment Adherence

Two studies sought to explore parameters of attachment and personality structure in patients at the beginning of treatment for SUDs. Given the high rates of drop-outs [e.g., Ref. (74)] and the often-discussed differences between SUD [e.g., Ref. (2)], the results of these studies may help improve treatment adherence and consequently treatment outcomes.

#### Study 4

As there is still some debate about whether various forms of SUD differ regarding their association with insecure attachment [e.g., Ref. (42)] and impairments in personality structure [e.g., Ref. (138)], we examined these parameters in inpatients with either a PUD or an Alcohol Use Disorder (AUD) in comparison to a non-drug-using control group (98).

##### Methods and Results

In the first study (98), 66 inpatients diagnosed with AUD, 57 inpatients diagnosed with PUD, as well as 114 non-drug-using control subjects (CS) completed the *Attachment Style Questionnaire* (ASQ) [Ref. (139, 140), and the *Borderline Personality Inventory* (BPI) (141).

Compared to CS, inpatients with AUD or PUD showed higher levels in every facet of borderline personality structure and the attachment facet “Relationships as Secondary” as well as lower levels in every other facet of attachment. These differences were especially distinctive in the area “Confidence in Self and Others” (*eta* *^2^* = .22), which indicates secure attachment and in the total amount of borderline pathology (*eta* *^2^* = .30), respectively. No differences could be observed between AUD and PUD inpatients. Separate correlation analyses revealed that attachment and personality structure were unrelated in each group.

##### Discussion of Results

While the results of this study (98) confirmed that SUDs are linked to deficient attachment (42, 100) and increased borderline pathology (100, 142), they furthermore indicate that impairments in those areas are similar for AUDs and PUDs. However, although psychodynamic theory closely links early attachment experiences to personality structure (7), no distinctive link between impairments in those areas could be observed. Interestingly, inpatients with a SUD showed lower levels in several facets of attachment deficiencies (e.g., Need for Approval) than healthy controls (140). However, we argue that lower and higher than average levels in these areas could be considered problematic as they might indicate rigid patterns in interpersonal experiences. Correspondingly, from a psychodynamic perspective, one of the major therapeutic aims is described as “greater flexibility in interpersonal relationships and an enhanced capacity to meet interpersonal needs” (p. 99) (143). The increased borderline pathology we detected in inpatients with an SUD (98) suggests that impairments in personality structure can be present independent of comorbid personality disorders. In line with research on the dual diagnosis of SUD and personality disorders (142), we therefore support a dimensional approach in the study and treatment of personality pathology with an SUD.

#### Study 5

Building on Study 4, a second study (102) focused on the role of attachment in treatment adherence during the first 6 weeks of a residential treatment program.

##### Methods and Results

One hundred twenty-two inpatients (34 female), diagnosed with AUD (*n* = 66) or PUD (*n* = 57), were tested at treatment entry. After 6 weeks, the 47 inpatients remaining in treatment were tested for a second time. Both times participants completed the ASQ [Ref. (139, 140).

Using all ASQ subscales, agglomerative cluster analysis on the total sample suggested a two-cluster solution: Cluster I was defined by higher scores in “Confidence in Self and Others,” while Cluster II was defined by higher scores in “Need for Approval” and “Relationships as Secondary.” Further analyses showed that inpatients in Cluster I were more likely to drop out of treatment during the first 6 weeks. In hierarchical regression analyses predicting treatment adherence, with the control variables sex and psychiatric comorbidity at Step 2, attachment security (Cluster I vs Cluster II) added approximately 6% of variance at Step 3.

##### Discussion of Results

The results of the fifth study (102) indicate that self-reported secure attachment might be linked to lower treatment adherence in patients with SUDs. This unexpected finding might be attributed to the influence of self-reflection, with a lower ability for self-reflection resulting in more secure self-appraisal but also to an increased likelihood of treatment drop-out. In line with this, self-report measures of adult attachment—in comparison to attachment interviews—are considered to be more likely influenced by distorted self-images while insufficiently assessing repressed information (144).

Consequently, self-reported attachment security may be attributed to an idealized self-view defined by primitive defense mechanisms (e.g., splitting or denial) (102). Furthermore, our findings potentially reflect a unique attribute of therapeutic communities (124) that threatens such narcissistically distorted self-appraisals: In patients with this form of self-appraisal, the high amount of group cohesion potentially leads to increased cognitive dissonances that consequently increase the likelihood of treatment drop-out (123). The reduction of narcissism in the therapeutic community might also explain the decrease in Confidence in Self and Others after 6 weeks of treatment (102). This is also part of the concept of the therapeutic community itself, as patients are encouraged to explore their interpersonal deficits (124). Furthermore, the decrease in Confidence in Self and Others likely also mirrors the decline of an initial euphoria experienced when entering treatment and being sober after severe substance use (102).

## Conclusion and Outlook

While the application of attachment theory always implies a developmental approach, this article focused on the basis from which individuals diagnosed with a PUD might progress towards recovery. Furthermore, although research focused on attachment already contributed important insights into the characteristics of our social nature, “an important enterprise for the future is to consider how attachment is differentiated from, and integrated with, other features of development” (p. 25) (145).

Consequently, the characteristics and treatment requirements connected to PUDs especially need to be addressed in more detail, as this diagnosis is highly common in individuals seeking treatment while simultaneously being associated with poor treatment success [e.g., Ref. (2)]. Several important conclusions regarding SUD—particularly PUD—and their treatment can be drawn from the original research presented above.

### Implications for Clinical Practice

Although the completion of treatment is closely linked to favorable treatment outcome, it is more likely for a patient to drop out of treatment than to complete it: According to a systematic review by Brorson and colleagues (146), the most consistent risk factors for dropping out were cognitive deficits, younger age, personality disorders, and low treatment alliance. Conversely, the effects of treatment are dose related: While more and longer treatments usually lead to a better outcome, disruptions in attachment to the program or the clinical staff increased the likelihood of relapse and drop out (73). Considering the largely insecure attachment status of inpatients with PUD (98–102) may consequently improve treatment adherence.

As mentioned above, the primary function of adult attachment relationships may be seen in the regulation of emotions (38). The regulation of emotions through social interactions is a key function of the attachment system, as the quality of the attachment bond influences emotional functioning and regulation capabilities as well as styles of interpersonal relating from childhood on into adulthood (46, 147). While impairment in cognitive reappraisal, an explicit emotion regulation strategy, seems to be relevant in PUD (101), implicit, i.e., automatic and largely unconscious emotion regulation strategies, may be of even greater relevance, as they are likely more closely connected to the mental representations of self and others included in attachment and personality structure (148).

Furthermore, future studies on PUD may also consider the connections between boredom and substance use: Boredom, which is connected to an emptiness stemming from social isolation as well as a lack of attachment to others (149), represents a critical factor in relapse (149) among others through its connection to increased risk-taking behaviors (150). Boys and colleagues (151) found that close to 90% of young (16–22 years) poly-substance users consumed illicit substances to enhance an activity with 83% consuming the substances to decrease boredom.

Interestingly, while the studies on PUD described in detail above (98–102) focused on inpatients in therapeutic communities, the relation between attachment and psychological distress can also be found in SUD outpatients (152): Here, an insecure attachment is again more common than in healthy controls. Furthermore, fearful attachment appears to be associated with higher levels of psychological distress. Importantly, psychological treatments with a directive, reflective, or supportive orientation appear to result in more patients having a secure attachment style by the end of treatment (152).

While the consideration of different emotions and their role in SUD is of great importance, the absence of these emotions or of their perception has to be considered as well: For example, a recent study (153) found that insecure attachment also seems to be associated with dissociation and alexithymia in individuals with SUD. As they inhibit the identification and verbalization of emotions, dissociation and alexithymia also impair the communication with others and thus the mutual understanding (153).

A change from insecure to secure attachment style might therefore be considered an important goal in SUD treatment, as it could prevent patients from applying defense strategies involving substance use to regulate their emotions and interpersonal relationships (152).

### Implications for Research

Given that a meta-analytic review by Dutra and colleagues (74) found that individuals with PUD—compared to other SUD—appear to profit the least from treatment interventions, more research on the characteristics and treatment requirements of individuals with PUD is still very much needed. Therein, attachment theory provides a bio-psycho-social model for human behaviors and experiences in relation to the regulation of stress and emotion in social situations (154). Following this approach, the presented results (98–102) underline the conceptualization of PUD as an “Attachment Disorder” as well as the value of the bio-psycho-social perspective in this research area. As the influence of attachment always has to be considered in the context of other risk factors (13), exploring and integrating the clinical characteristics of individuals with PUD are of vital importance for future research on treatment approaches. For example, polydrug use can frequently be found in connection to sexual behaviors (155–158). However, few studies to date seem to have explored the role of sexual behaviors in PUD (159) and no study seems to have explored their romantic relationships.

In addition. the above described studies on inpatients with PUD (99, 100) highlight the fact that insecure attachment and other behavioral impairments in inpatients with PUD are paralleled by extensive impairments in white matter integrity (99, 100), most notably in tracts connected to facets of emotion regulation (e.g., impaired decision-making and higher risk taking behavior) (69, 113). Consequently, a potential neuroplasticity during the treatment of SUD in general—and the long-term stay in a therapeutic community in particular—should be explored in future research.

Furthermore, the results of the above described studies (98, 100, 101) suggest that future research on the treatment for SUD would benefit from the assessment of personality structure and related psychodynamic interventions. Therein, Kohut’s (160) theory that a specific substance can be seen as a “replacement for a defect in the psychological structure” matches with the widespread impairments in personality structure found in inpatients with PUD (101). Furthermore, the influence of traumatic experiences in childhood on the amount of addictive behaviors displayed in young adulthood (161) seems to be mediated by impairments in personality structure and insecure attachment (162–164).

While the above described studies on inpatients with PUD focused on primary emotions and certain emotion regulation strategies (99, 101), there is vast potential for additional contributing factors in PUD related to emotions and their regulation. Among emotion regulation processes, social affect regulation can be seen as a bottom-up mechanism, while affect regulation without support from others can be seen as a top-down mechanism. These top-down mechanisms include effortful cognitive and attentional emotion regulation strategies, such as suppression or cognitive reappraisal, that rely heavily on the prefrontal cortex (46). Consequently, an examination of the neural correlates of bottom-up mechanisms in PUD would generate further important insights.

The importance of research on SUD—and especially PUD—as well as the need for evidence-based effective treatment strategies is further underlined by the consideration of transgenerational effects: For example, in a recent study by Tuhkanen and colleagues (165), only 7% of infants born to mothers with recent or current substance use showed no neurological impairments during their first days of life.

### Limitations

As different definitions of polydrug use are applied in SUD research, the comparison and integration of results can be difficult (87). These tendencies may lead to research results that provide little relevant information for clinicians involved in SUD treatment programs. On the other hand, explicit and systematic evaluations of polydrug use could have a high clinical as well as public health relevance (60). Consequently, the possibility of concurrent use of other substances should always be taken into consideration, even when only the use of one particular psychotropic substance is the focus of a study (57). For example, while polydrug use is often considered a barrier in the prevention of hepatitis C virus (HCV) transmission in individuals who inject prescription opioids, research on the underlying mechanisms is relatively sparse (166).

Furthermore, the results of different studies on attachment in adults are also often hard to compare or to summarize (148). In general, self-report measures—as used in the presented studies (98–102)—are thought to be limited in their ability to assess all areas of attachment patterns, as they solely rely on conscious attitudes and behaviors (144). However, they are also considered to be more focused on current attachment patterns in various relationships, while the Adult Attachment Interview (167) solely focuses on the relationship with the parents.

As inpatient participants in the described studies were enrolled in a therapeutic community at the time of data acquisition (98–102), this may have influenced the results in various areas. Among others, the decline in reported attachment security after the initial treatment phase (102) could also—at least partly—be attributable to this treatment approach. However, to date, hardly any empirical studies have investigated the role of attachment theory and related parameters (e.g., therapeutic alliance) for the conceptual framework and success of the therapeutic community [e.g., Ref. (168)]. Consequently, further research is very much needed to explore these mechanisms.

The high levels of comorbid mental disorders in inpatients with SUD in general [for an overview, see Ref. (169)] and PUD in particular also have to be considered in the interpretation of research results. Conversely, deficiencies in emotion processing and regulation are a known “liability spectrum that underlies many different mental disorders” (p. 154) (36). Internationally, this high risk of co-occurrence appears in both directions: While between 40% and 50% of individuals with a SUD also have at least one other psychiatric diagnosis, other psychiatric diagnoses also show a high rate of comorbid SUD [for an overview, see Ref. (169)]. The question of how co-occurring psychiatric disorders influence the participation and outcome of treatment in SUD has also not yet been fully answered (170). Overall, treating co-occurring affective and personality disorders as diagnoses in their own right generally seems to lead to better outcomes than only treating SUD and an integrated treatment approach can therefore be considered evidence based (170).

In line with this, maltreatment, and especially cumulative abuse, during childhood is associated with several related mental disorders, including SUD and PUD (171, 172). Therefore, a more extensive assessment of traumatic experiences might reveal different profiles among individuals with SUD that could profit from different treatment strategies.

While our research highlights the presence of white matter impairments in inpatients with PUD (99, 100), we did not specifically investigate possible influences of the number of abused substances or the intensity of abuse. This may be an interesting area for future research, as recent findings suggest that some neural impairments may be related to specific substances while others are related to the amount of poly drug use (173).

Lastly, many different approaches may have been taken to explore the current theoretical and empirical literature on PUD from an attachment perspective. Therein, different types of reviews are known to have specific strengths and weaknesses (174).

### Future Directions

The focus on PUD is now more important than ever, given that the diversification of certain products (e.g., nicotine, marijuana, prescription drugs) in recent years seems to have contributed to an increased polydrug use in adolescents (88, 89). In turn, polydrug use is strongly associated with later SUD and related health issues (175, 176).

The studies on PUD described above and in additional publications (98–102) clearly highlight the importance of attachment and related parameters in PUD as well as their bio-psycho-social integration. Future studies might more closely focus on the influence of attachment-based interventions on emotion regulation abilities as well as a potentially related neuroplasticity.

While recovery represents an important paradigm in the treatment of SUD, the definition of recovery has been extended beyond a reduction in use or sustained abstinence and now also includes the enhancement of global well-being as well as a reintegration into a prosocial community. To date, few studies have incorporated this broadened definition of recovery into their design (79). The most commonly used outcome measure, i.e., treatment retention and abstinence from the primary psychotropic substance (177), might not be able to fully assess the effects of psychosocial interventions, e.g., changes in emotion regulation and other attachment parameters. Especially in long-term inpatient PUD treatment settings (e.g., therapeutic communities), neuroplastic changes, which are still rather unexplored, might represent important additional parameters for the assessment of treatment outcomes.

## Author Contributions

MH-R and H-FU conceptualized the study. MH-R wrote the draft. H-FU proofread the manuscript. Both authors gave their consent for publication.

## Conflict of Interest Statement

The authors declare that the research was conducted in the absence of any commercial or financial relationships that could be construed as a potential conflict of interest.
